# First‐in‐human outpatient management of recurrent NMIBC with the Multi4 instrument

**DOI:** 10.1111/bju.70445

**Published:** 2026-09-11

**Authors:** Miden Melle‐Hannah, Henrik Kjölhede, Henrik Bäckdahl, Martin E. Johansson, Joakim Håkansson

**Affiliations:** ^1^ Multi4 Medical AB Gothenburg Sweden; ^2^ Department of Urology, Institute of Clinical Sciences Sahlgrenska Academy, Gothenburg University Gothenburg Sweden; ^3^ Department of Urology Sahlgrenska University Hospital Gothenburg Sweden; ^4^ Department of Clinical Pathology Sahlgrenska University Hospital Gothenburg Sweden; ^5^ Sahlgrenska Center for Cancer Research University of Gothenburg, Sahlgrenska Academy Gothenburg Sweden; ^6^ Department of Laboratory Medicine, Institute of Biomedicine University of Gothenburg, Sahlgrenska Academy Gothenburg Sweden; ^7^ RISE Research Institutes of Sweden Life Science, Medical Device and Diagnostics Borås Sweden

**Keywords:** bladder cancer, non‐muscle invasive bladder cancer, transurethral resection of bladder tumour, cystoscopy surgery, outpatient management

## Abstract

**Objectives:**

To evaluate the feasibility, safety and diagnostic performance of outpatient management of recurrent non‐muscle‐invasive bladder cancer (NMIBC) under local anaesthesia using the Multi4 instrument, a series of preclinical studies were performed followed by a first‐in‐human clinical investigation. NMIBC is associated with frequent recurrence, requiring repeated transurethral resection of the bladder (TUR‐B) under anaesthesia, resulting in substantial healthcare burden. The Multi4 instrument integrates local anaesthesia injection, diathermy biopsy, coagulation and automated tissue retrieval. The primary objective was to determine whether Multi4 provides histologically evaluable biopsies in an outpatient setting, and secondary objectives included safety, usability, patient‐reported outcomes and health‐economic impact.

**Methods:**

Preclinical *in vivo* evaluation was performed in 13 pigs to assess safety, biopsy quality, coagulation efficacy and perforation risk. A prospective, open‐label, single‐arm clinical investigation enrolled 18 adults with recurrent Ta/T1 NMIBC (≤10 mm lesions). Procedures were performed under local anaesthesia using flexible cystoscopy. The primary endpoint was the proportion of evaluable biopsies. Secondary endpoints included adverse events, usability, pain (0–10 scale), EQ‐5D and cost comparison versus standard TUR‐B. Diagnostic yield was also assessed at the patient level.

**Results:**

A total of 550 porcine biopsies demonstrated immediate haemostasis and preserved tissue architecture suitable for histological assessment. Perforation occurred after 7–11 sequential biopsies at the same location, indicating a wide safety margin. Clinically, 134/145 biopsies (92%) were evaluable and the primary endpoint was met. Diagnostic yield per patient was 89%. No device‐related adverse events or Clavien–Dindo complications occurred. All procedures were completed under local anaesthesia without conversion to standard TUR‐B or use of additional instruments. The median pain score was 0.5/10. Estimated per‐procedure cost reduction was 73–89% versus TUR‐B.

**Conclusions:**

Multi4 enables feasible and safe outpatient biopsy and lesion management of small recurrent NMIBC under local anaesthesia with substantially reduced resource use while maintaining diagnostic reliability.

**Abbreviations**
AE: adverse event
BC: bladder cancer
CPP: Cost Per Patient
EQ‐5D: EuroQol 5‐Dimension
FAS: full analysis set
NMIBC: non‐muscle‐invasive bladder cancer
QoL: quality of life
TUR‐B: transurethral resection of the bladder
UTI: urinary tract infection

## Introduction

Bladder cancer (BC) is the tenth most frequently diagnosed malignancy worldwide [1], Incidence rates are higher in the European Union, reaching 21–27 per 100 000 men and up to 5.8 in women [2]. The disease is characterized by a high recurrence rate of up to 78% [3], and many patients experience multiple recurrences [4]. BC can progress to invasive disease [5, 6], requiring long‐term surveillance and repeated interventions that place a substantial burden on healthcare systems.

Approximately 75% of newly diagnosed cases are non‐muscle‐invasive BC (NMIBC), confined to the mucosal or submucosal layers [7, 8, 9]. Early and accurate diagnosis is essential, as delays in transurethral resection of the bladder (TUR‐B) are associated with more advanced tumours, reduced outcomes and lower survival [10, 11, 12, 13]. TUR‐B remains the standard of care for NMIBC and is typically performed under general or regional anaesthesia to remove visible tumour tissue and obtain diagnostic samples [7, 14, 15, 16]; however, it requires preoperative preparation, anaesthesia and hospitalization, imposing high resource demands [17].

For selected patients with small, low‐grade recurrences, office fulguration under local anaesthesia may be used [18], but cold‐cup biopsies taken in this setting cause bleeding, impair visibility and may compromise diagnostic quality [19]. Despite widespread use, the basic tools for bladder tumour resection have changed little over decades. The narrow cystoscope channel restricts instrument size, forcing surgeons to alternate between devices for anaesthesia, biopsy, coagulation and tissue retrieval [20, 21]. To overcome these limitations, we developed the Multi4 instrument, a multifunctional device that integrates local anaesthesia injection, diathermy‐based biopsy, coagulative diathermy and automated biopsy transport. Operable through a flexible cystoscope, it enables diagnostic and therapeutic procedures entirely under local anaesthesia in a single outpatient visit. This study presents preclinical and clinical results demonstrating its feasibility, safety and potential to streamline NMIBC management.

## Methods

A detailed description of the *ex vivo* functionality testing and health‐economic analysis is available in the Supporting Information.

### Device Description

The Multi4 system (hereafter referred to as the Multi4 instrument) includes a single‐use, endoscopic instrument, a connected pump and a container for collecting the biopsies. The device is designed to integrate four functions: (i) injection of local anaesthetics via a fine needle, (ii) tissue‐cutting forceps with electrocautery, (iii) automated biopsy transport and collection through fluidic transport, and (iv) coagulation for haemostasis and tissue ablation (closed forceps). The flush function (pressing a pedal connected to the device) allows the clinician to flush approximately 16 mL in 5 s. The device can be used to excise the whole tumour or take a few biopsies and fulgurate the remaining tissue. The device is connected to a monopolar energy source for cutting and cautery and therefore uses minimal conductive fluid, such as sterile water and a dispersive return electrode (grounding pad).

### *In Vivo* Safety and Functionality Evaluation in Pigs

All animal experiments followed ARRIVE 2.0 guidelines and were conducted in compliance with the Swedish guidelines for the care and use of laboratory animals (Directive 2010/63/EU). The protocol was approved by the regional animal ethics committee, Gothenburg, Sweden (Dnr 2721/2019). Detailed procedures are provided in the Supporting Information.

### Clinical Performance and Safety Study

#### Study Design

This prospective, pre‐market, open‐label, single‐arm clinical investigation was conducted to evaluate the performance and safety of the Multi4 instrument in adult patients with suspected recurrence of NMIBC. As a first‐in‐human feasibility investigation, the sample size was determined pragmatically based on expected recruitment and regulatory guidance for early device evaluation rather than a formal statistical power calculation. The study corresponds to IDEAL stage 2a (development) for surgical innovation and is reported accordingly and was conducted in accordance with ISO 14155:2020 guidelines for clinical investigations of medical devices. The study was approved by the Swedish Ethical Review Authority and the Swedish Medical Products Agency (5.1‐2023‐61 059) and was registered at ClinicalTrials.gov (NCT06136312).

#### Participants

Inclusion criteria for eligible participants were patients aged ≥18 years with a documented history of Ta/T1 G1–3 BC and visible recurrence ≤10 mm on cystoscopy. Key exclusion criteria included pregnancy, current urinary tract infection, and the presence of an implantable cardioverter defibrillator that could not be inactivated. Informed consent was obtained from all participants. None of the tumours were clinically suspected to be invasive. For patients with invasion of the lamina propria, a repeated TUR‐B with conventional technique was performed.

#### Procedure

Cystoscopy was performed to locate the lesion, the bladder was filled with sterile water and the Multi4 instrument was applied according to its instructions for use. Local anaesthesia (lidocaine 10 mg/mL, Accord Healthcare) was injected into the bladder wall at the tumour base. The lesion was resected in multiple fragments using the electrocautery and tissue collection functions of the Multi4 instrument, followed by coagulation of the resection area to achieve haemostasis and minimize the risk of local recurrence. This technique corresponds to standard TUR‐B, where tumours are fragmented for removal. After the procedure, patients were observed for about 30 min and vital signs and urination were monitored, including assessment for haematuria.

#### Follow‐Up and Data Collection

Patients were observed for at least 30 min after the procedure and completed a pain/discomfort numerical rating scale (0 to 10) before discharge. A follow‐up phone call was conducted 7 ± 5 days after the procedure to assess adverse events (AEs), satisfaction and quality of life (QoL) using the EQ‐5D questionnaire.

#### Endpoints

The primary endpoint was the proportion of evaluable biopsy samples, assessed by a pathologist using pre‐defined histological quality parameters.

Secondary endpoints included:

- procedure‐related safety (AEs and complications)
- system performance in outpatient settings
- usability and operator satisfaction
- patient‐reported outcomes (pain, QoL)
- Preliminary assessment of potential health‐economic benefits.

#### Statistical Analysis

Because multiple biopsies were obtained per patient, the patient was considered the primary clinical unit for diagnostic performance analyses. The primary endpoint was tested using a one‐sided exact binomial test (*α* = 0.025) with the null hypothesis *H*_o_: π < 50%. A conservative feasibility threshold of 50% evaluable biopsies was selected for this exploratory first‐in‐human investigation, reflecting the absence of prior clinical performance data for the device.

## Results

Results from the *ex vivo* functionality testing are presented in the Supporting Information.

### Safety and Functionality Evaluation *In Vivo* in Pigs

As no established animal model for urinary bladder tumours exists, the safety and functionality of the Multi4 instrument were evaluated in porcine bladders. *In vivo* testing was performed in 13 female pigs (65–70 kg). A flexible cystoscope was inserted transurethrally and the Multi4 instrument advanced through its working channel (Fig. 1a). The device operates in three positions (Fig. 1b): (i) needle extended with open forceps for local anaesthetic injection, (ii) needle retracted with open forceps for biopsy acquisition, and (iii) closed forceps for biopsy transport or coagulation. The injection needle was tested at multiple sites by injecting 3 mL of saline (NaCl) into the bladder wall, producing a localized tissue bolus that facilitated subsequent biopsy sampling (Fig. 1c,d). To evaluate the performance of the diathermy‐based biopsy forceps (Fig. 1c, e), more than 550 biopsies were obtained from all bladder regions, including the dome, base and anterior, posterior, and lateral walls. A biopsy was taken by opening the forceps, grasping tissue with a handle button and activating a foot pedal to deliver a diathermy pulse that excised tissue within the closed forceps. No bleeding occurred after any of the >550 biopsies. A second pedal activated the integrated pump, automatically transporting each specimen through the instrument into a sealed collection container (Fig. S1).

**Fig. 1:**
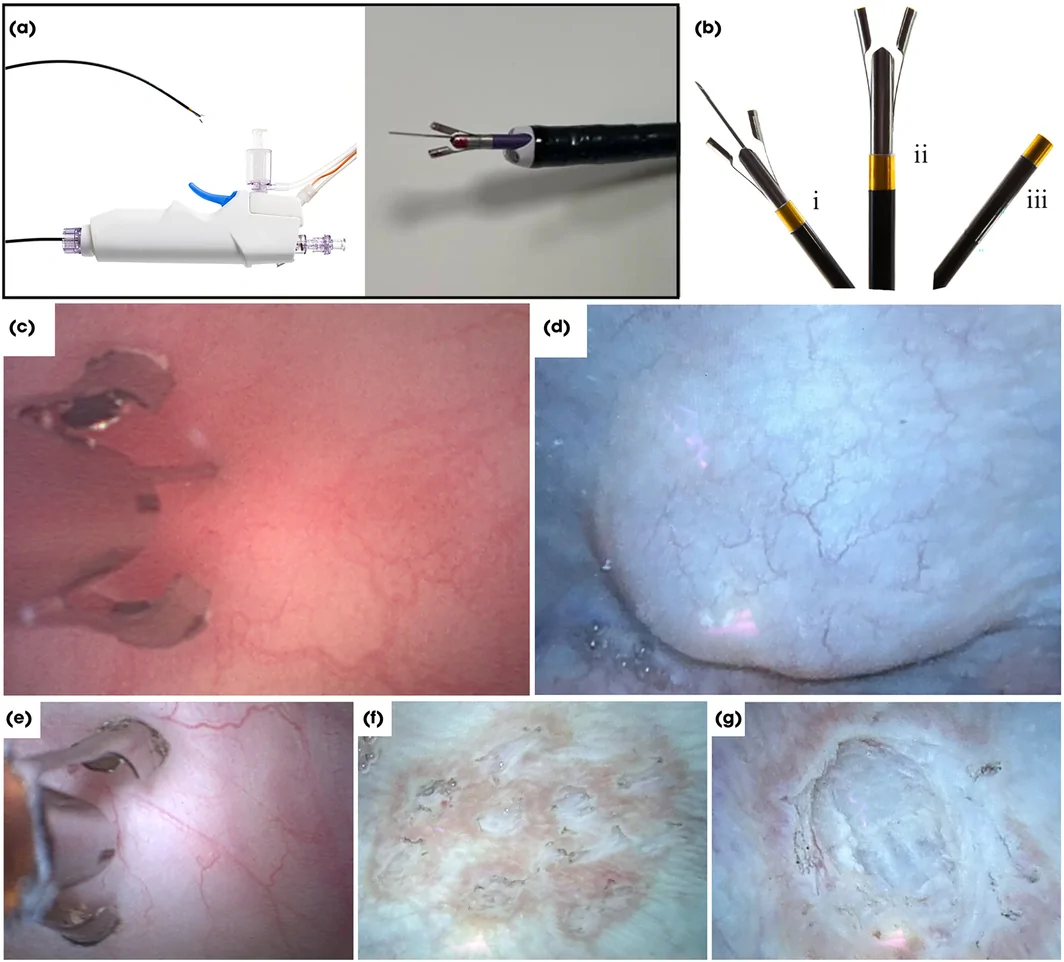
The Multi4 instrument inserted through a cystoscope and demonstration of its injection needle and biopsy functions. **(a)** The Multi4 instrument with the handle (left). The Multi4 instrument advanced through the working channel of a flexible cystoscope (right). **(b)** The distal end of the Multi4 instrument operating in three distinct modes: (i) with the forceps open and the needle centrally positioned extended for administering local anaesthetic; (ii) with the needle retracted and the forceps open to enable tissue sampling, which is completed by activating the handle button to close the forceps; and (iii) with the forceps closed and the needle retracted, allowing for transportation of the biopsy through the instrument to a sample container, or use of the coagulation function. **(c)** *In vivo* image of the injection needle inserted into the bladder wall of a pig. **(d)** Formation of a localized tissue bolus following injection of saline into the bladder wall. **(e)** The biopsy forceps of the Multi4 instrument, shown in the open position, ready to grasp bladder tissue. **(f)** Individual biopsy sites within a defined region of the bladder wall. **(g)** A cavity in the bladder after six sequential biopsies taken from the same location.

To mirror clinical practice, biopsies were taken both as single samples (Fig. 1f) and as a series of five to six sequential samples from the same site (Fig. 1g). All biopsies were consistent in size and histological quality across bladder regions.

As a reference, 50 biopsies were taken using standard cold‐cup forceps, which mechanically tear tissue from the bladder wall. Bleeding occurred at all biopsy sites (Fig. 2a). The diathermy coagulation function of the Multi4 instrument was then assessed for haemostatic efficacy. By closing the forceps (Fig. 1b(iii)), placing them on the bleeding site and activating the coagulation pedal, bleeding was completely stopped in all cases (Fig. 2b). Multiple biopsy sites after coagulation (Fig. 2c) show effective haemostasis. The diathermy function was also used for controlled ablation over a broader area (~1 cm^2^), simulating tumour eradication (Fig. 2d).

**Fig. 2:**
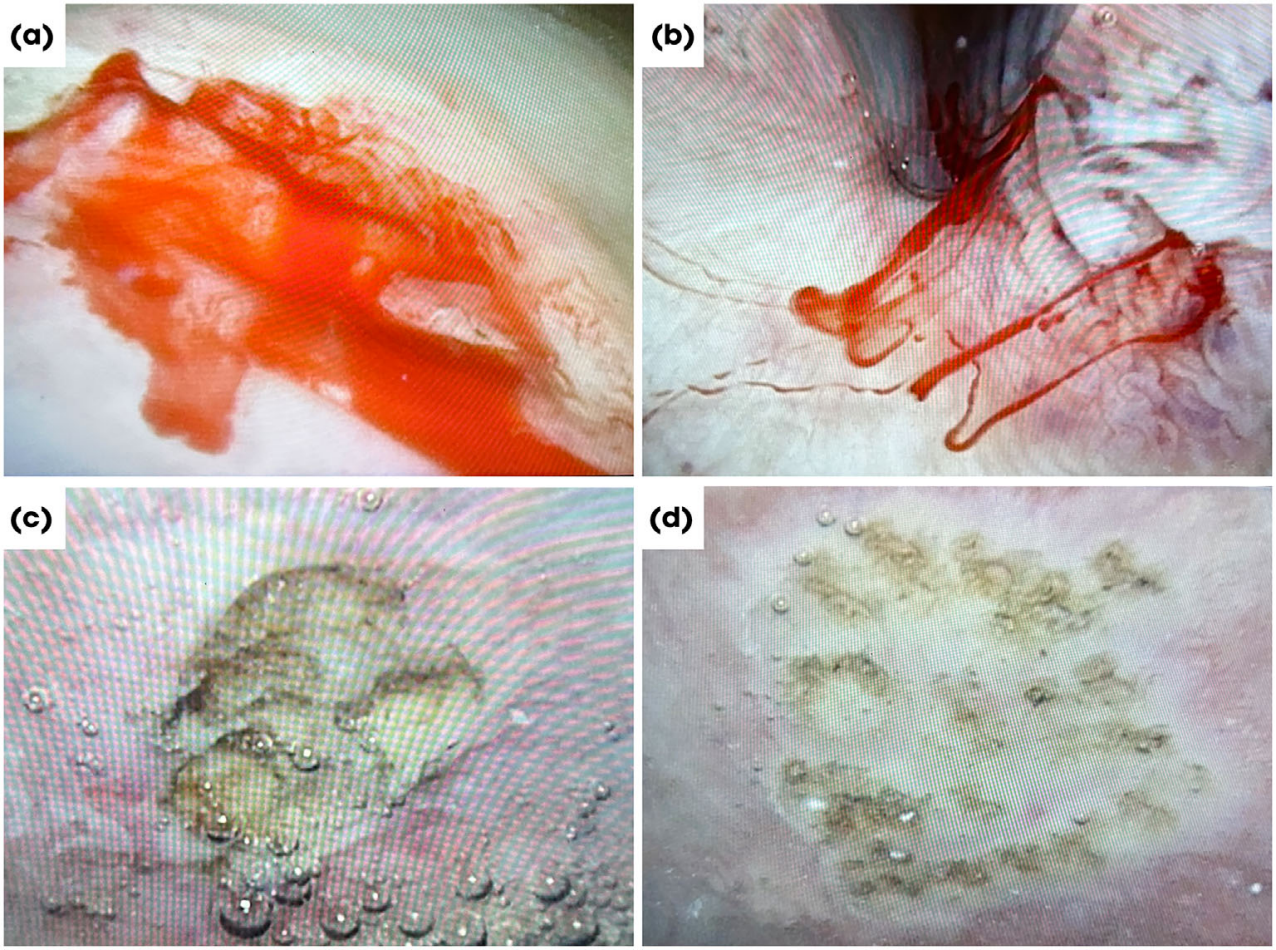
Haemostasis and tissue ablation using the coagulation function of the Multi4 instrument. **(a)** Bleeding observed after a biopsy performed with standard cold‐cup biopsy forceps. **(b)** Effective haemostasis achieved by activating the coagulation function of the Multi4 instrument with the forceps in the closed position. **(c)** Multiple biopsy sites after successful application of the coagulation function to control bleeding. **(d)** Bladder wall after application of the coagulation function over an area of approximately 1 cm^2^, simulating tumour tissue ablation.

To further assess haemostatic performance, biopsies were taken with cold‐cup forceps after intravenous administration of 10 000 IU heparin to induce anticoagulation. Despite active bleeding at the biopsy sites, the Multi4 coagulation function achieved complete haemostasis in all cases.

To evaluate the risk of perforation from repeated biopsies, sequential samples were collected from the same site until perforation occurred. Leakage of contrast medium, visualized by X‐ray, confirmed perforation. Testing in five pigs showed that, despite the thinner porcine bladder wall, perforation occurred only after 7–11 biopsies, indicating a wide safety margin.

### Clinical Performance and Safety Study

#### Study Population

Of the 22 enrolled patients, 18 completed the study per protocol and were included in the full analysis set. Four patients were excluded: two withdrew consent before the procedure, one had no visible lesion at cystoscopy, and one procedure was aborted because of technical malfunction. Participants ranged in age from 45 to 95 years (11 males, 7 females). Detailed patient‐level clinical characteristics, tumour features and biopsy outcomes are summarized in Table 1. All procedures were completed under local anaesthesia without the need for sedation or systemic analgesia.

**Table 1:** Patient‐level clinical characteristics, tumour features and biopsy outcomes in the full analysis set.

Patient no.	Pathology with Multi4	Intravesical instillations	Recurrence	Recurrence free follow‐up (months)
1	Benign	No	TaG1 at 6 months	
2	TaG1	No		24 months
3	CIS	BCG	Persistent CIS leading to radical cystectomy	
4	Benign	No		24 months
6	TaG3	BCG	CIS at 6 months, no recurrence at 12 months after gemcitabine/docetaxel	
7	Benign	No		18 months
8	TaG2	Mitomycin	Progression to T2 after 6 months, curative radiotherapy	
9	TaG2	Mitomycin		18 months
10	TaG2	No		18 months
11	TaG2	BCG		18 months
12	TaG1	No		18 months
13	TaG1	No		18 months
17	TaG1	No		12 months
18	TaG2	BCG	TaG3 at 12 months	
19	Benign	No		No follow‐up
20	TaG1	No	TaG1 at 6 months	
21	TaG1	Mitomycin	TaG1 at 4 months	
22	CIS	No	Immediate radical cystectomy	

BCG, Bacillus Calmette‐Guérin; CIS, carcinoma in situ; TaG, tumour stage/grade.

#### Biopsy Performance

A total of 153 biopsy attempts yielded 145 samples, of which 134 (92%) were evaluable, meeting at least three of the four parameters set as criteria for evaluability. The predefined primary endpoint (≥80% evaluable biopsies) was achieved with a 92% evaluability rate (*p* = 5.7 × 10^−34^). In 16 of 18 patients (89%), the biopsies allowed for a definitive BC diagnosis. The two remaining patients had clinically unclear lesions that showed inflammation in the biopsy. Fig. S2 illustrates the surgical treatment of a tumour using the Multi4 instrument.

Histological evaluation of biopsies obtained with the Multi4 instrument and conventional cold biopsy forceps from porcine bladder demonstrated equal tissue preservation quality and diagnostic utility. Biopsies taken with the Multi4 instrument in a clinical context resulted in larger and deeper specimens, facilitating pathological evaluation and diagnosis (Fig. 3).

**Fig. 3:**
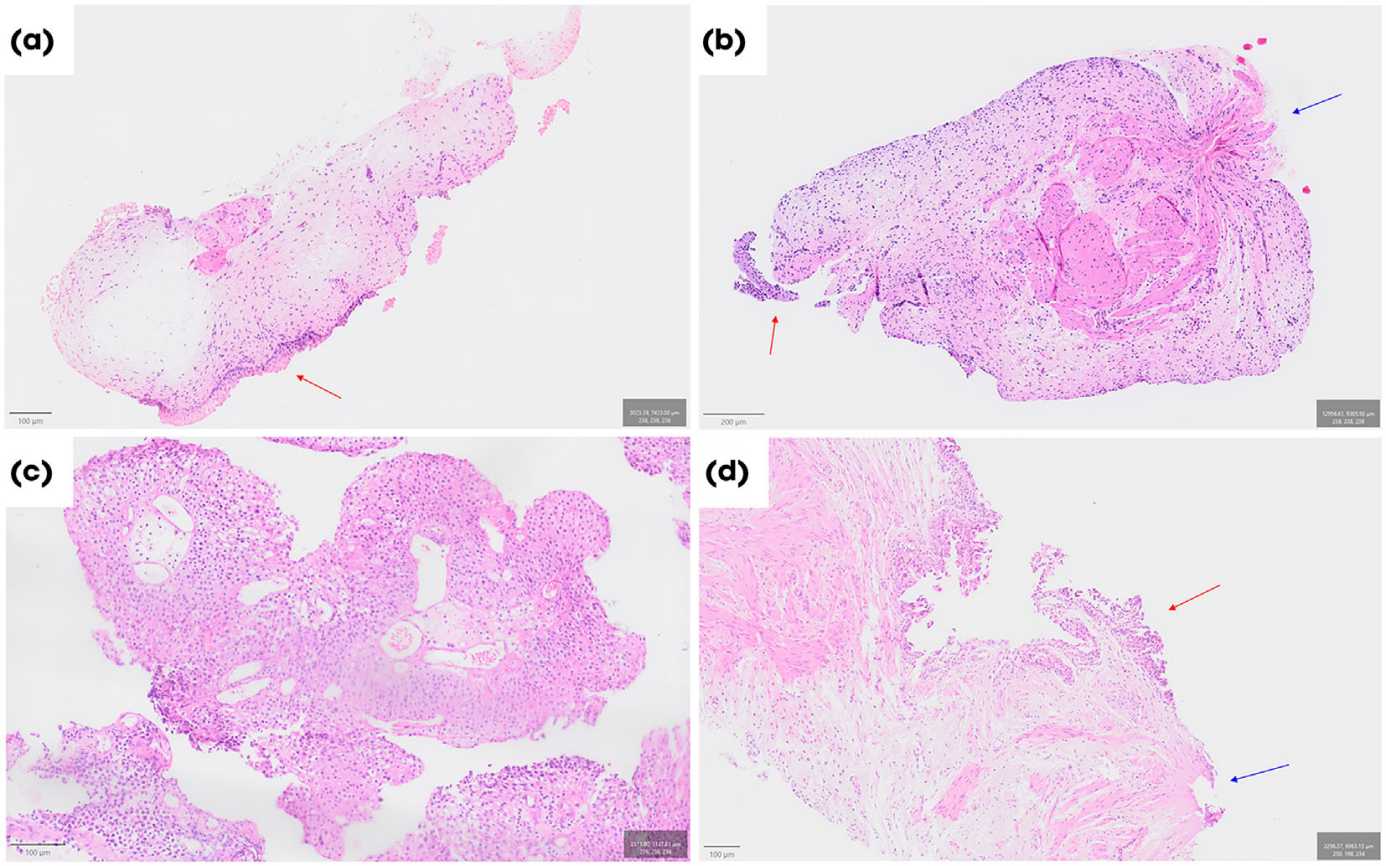
Haematoxylin and eosin‐stained histological sections from bladder wall and tumour. **(a, b)** Representative images of biopsies taken from the porcine bladder wall using **(a)** conventional cold biopsy forceps, where the red arrow indicates urothelium attached to the underlying stroma and **(b)** the Multi4 instrument, where the red arrow indicates dislocated urothelium unattached to the underlying stroma and the blue arrow indicates the pinched cautery effect of the Multi4 instrument. **(c, d)** Representative images of tumour biopsies taken with the Multi4 instrument in the clinical study. **(c)** One of six biopsies taken from a 1 cm recurrent papillary pTaG2 tumour. **(d)** Biopsy taken from a pTaG1 urothelial tumour. Only limited urothelium is present on the biopsy, indicated by the red arrow, but sufficient for diagnosis. The blue arrow indicates the pinched cautery effect of the Multi4 instrument.

#### Safety

No device‐related AEs or serious complications occurred. Two cases of asymptomatic bacteriuria were reported but were unrelated to device use. No Clavien–Dindo‐classified complications were observed, and no catheterization after any of the procedures was necessary. Minor bleeding was effectively managed using the coagulation function.

#### System Performance and Usability

The Multi4 instrument successfully completed all procedures, including biopsy collection and bleeding control. Minor intraoperative bleeding occurred in 11 patients and was effectively stopped using the coagulation mode. In nine cases, residual tumour tissue was ablated with the same function. Procedure duration ranged from 4 to 30 min.

Usability was rated as high; all procedures were completed with a single device, except one requiring replacement because of technical malfunction. Good endoscopic visibility was maintained during the whole procedure in 16 of 18 cases. In the two cases with momentary poor visibility, the flushing function of the instrument was used to improve vision enough to coagulate the bleeding.

#### Patient‐Reported Outcomes

In total, 16 patients completed QoL assessments before and 1 week after the procedure. Results were favourable, with low pain scores (median 0.5 on a 0–10 scale) and stable or improved QoL. All patients reported feeling well after the procedure, and all (except one non‐respondent) stated they would choose Multi4 over the resectoscope for future treatment.

Fig. S3 compares the standard TUR‐B workflow with the Multi4 approach, illustrating how the new procedure eliminates preoperative visits, reduces staffing needs, replaces regional or general anaesthesia with local anaesthesia, shortens the procedure time from 30–90 min to ≤30 min, and removes the need for postoperative hospitalization.

#### Health‐Economic Analysis

A health‐economic analysis was performed by an independent third party using the Cost Per Patient (CPP) database at the Sahlgrenska University Hospital for 2023–2024, supported by data from the Orbit surgical planning system and supplemented with expert opinion. The mean total cost per standard TUR‐B was estimated at €2501 for outpatient care and €6625 for inpatient care (the average hospital stay was 1.7 days), including preoperative visit costs. In contrast, the cost for a Multi4 procedure was estimated at €663. This corresponds to cost reductions of approximately €1837 (73%) and €5965 (89%) per procedure, respectively, compared with outpatient and inpatient TUR‐B. In the most favourable scenario (20% lower costs with Multi4), the total procedure cost dropped to €530; in the least favourable scenario (20% higher), it rose to €795. Both scenarios still resulted in 79% reduced costs at the best and 68% reduced costs at the least for outpatient procedures and 92% reduced costs at the best and 88% reduced costs at the least for inpatient procedures.

A sensitivity analysis showed that the Multi4 procedure could increase in cost by up to 240% and still be more economical than standard outpatient TUR‐B. Break‐even occurred at a 280% cost increase.

## Discussion

This study demonstrated a new technological approach for outpatient diagnosis and treatment of recurrent BC using the multifunctional Multi4 instrument. The study combined preclinical validation with a first‐in‐human evaluation, providing a translational pathway from bench to clinic. The Multi4 device addresses the persistent shortcomings of conventional TUR‐B, including procedural complexity, bleeding and dependency on anaesthesia and inpatient resources. Integrating four key functions (local anaesthetic injection, biopsy, coagulation and automatic tissue retrieval) within a single flexible cystoscopic instrument enables efficient, safe and patient‐centred management of NMIBC under local anaesthesia.

*Ex vivo* testing established the technical reliability of the system and verified its precision in cutting and coagulation. More than 5000 tests confirmed reproducible energy delivery and consistent biopsy transfer, providing a stable platform for optimization before *in vivo* studies. Although the *ex vivo* model was performed in porcine bladders, it captured the key mechanical features of the human organ and permitted rapid iteration of design parameters in a cost‐efficient and ethical manner.

*In vivo* evaluation in pigs further confirmed system performance and procedural safety. The porcine bladder is widely accepted as the closest large‐animal analogue to the human organ [22, 23, 24, 25, 26, 27], with comparable dimensions and tissue composition that allow realistic cystoscopic manipulation. Despite the thinner, less fibrotic bladder wall of young pigs, sequential biopsies could be performed safely without perforation until 7–11 repetitions, indicating a generous safety margin. Haemostasis was immediate, even under heparinization, demonstrating effective energy control. Although hormonal variation and tissue fragility in young animals can influence histologic appearance, such as epithelial detachment (Fig. 3b), these differences were well recognized during interpretation and did not compromise the assessment of functionality or safety.

The main focus of the investigation was clinical translation. Procedures were performed entirely under local anaesthesia using a flexible cystoscope, confirming true office‐based feasibility. The Multi4 instrument incorporates an integrated injection needle that enables administration of local anaesthetic directly adjacent to the lesion. In the present study, all patients received peri‐tumoral lidocaine and tolerated the procedure well, as indicated by the very low pain scores. Our local experience is that cold cup biopsy and fulguration with separate instruments can be performed without local anaesthetics but with more discomfort. Whether this can be translated to the Multi4 device remains to be investigated. Biopsy evaluability reached 92%, exceeding the predefined feasibility threshold, and diagnostic yield was 89%, establishing that Multi4 provides tissue samples of sufficient depth and quality for histopathologic evaluation. This evaluability rate approaches those reported for conventional TUR‐B biopsies, which are typically in the range of 90–98%. Diathermy‐assisted biopsy preserved tissue architecture and minimized bleeding, maintaining visibility throughout the procedure. The immediate switch to coagulation after biopsy prevented impairment of the visual field, a limitation in cold‐cup methods. Deep and well‐oriented specimens (Fig. 3c,d) enabled accurate grading and staging, supporting diagnostic reliability. The present study was not designed to assess oncological outcomes such as recurrence rate, residual tumour rate or progression, and subsequent confirmatory TUR‐B was not systematically performed. Therefore, conclusions regarding therapeutic equivalence to TUR‐B cannot be drawn. Larger controlled trials with long‐term cystoscopic follow‐up are required to determine the oncological efficacy of the Multi4 approach. The study sample size was limited and not based on a formal power calculation, which is consistent with its exploratory, first‐in‐human design. The elimination of general anaesthesia and hospital admission markedly reduced patient burden. Reported pain scores were low, and almost all participants preferred the Multi4 procedure over standard TUR‐B. Surgeons reported intuitive operation, short learning curves, stable visibility and a typical procedure time within 30 min. These results indicate that Multi4 may represent a potential outpatient alternative for selected diagnostic and therapeutic procedures in recurrent NMIBC, enabling rapid diagnosis and same‐day discharge.

The workflow comparison (Fig. S3) highlights how the Multi4 approach condenses the multistep TUR‐B process into a single outpatient encounter. This transformation parallels shifts seen in other surgical specialties where technological miniaturization and energy integration have relocated procedures from operating theatres to clinics. Importantly, Multi4 does not compromise diagnostic accuracy or procedural safety but does achieve major gains in efficiency.

From an economic standpoint, BC remains among the costliest malignancies to manage because of its high recurrence rate and need for lifelong surveillance [6, 28, 29]. Botteman et al. reported BC as the most expensive cancer from diagnosis to death in the United States [30]. The present health‐economic analysis supports these observations and demonstrates that replacing conventional TUR‐B with Multi4 could reduce per‐procedure costs by 73–89%. Savings arise from shorter operative times and elimination of anaesthesia, preoperative consultations and inpatient care. Even under conservative cost assumptions, the Multi4 procedure remained economically favourable, with a wide break‐even margin, confirming its robustness and scalability for large‐volume implementation.

In conclusion, the Multi4 instrument represents a major technological advance in NMIBC management. The combination of local anaesthesia delivery, biopsy, coagulation and automated specimen retrieval into a single flexible cystoscopic device enables safe, effective and fully outpatient treatment. The system simplifies workflow, reduces healthcare costs and improves patient comfort without compromising diagnostic precision or safety. These results suggest a paradigm shift from hospital‐based to office‐based management of recurrent NMIBC. Future multicentre and randomized studies should confirm long‐term oncologic outcomes and further define its role in routine clinical practice.

## Author Contributions

All authors contributed to the study design. MM‐H, HK, ME‐J and JH performed experiments and analysed results. All authors contributed to data production, interpretation and writing of the manuscript.

## Disclosure of Interests

MMH is employed by Multi4 Medical AB, which holds the patent for the Multi4 instrument and partially financed the study. The remaining authors declare no competing financial interests.

## Funding

This study was supported by the strategic innovation programmes Swelife and Medtech4Health, a joint initiative by Vinnova, Formas and the Swedish Energy Agency (contract no. 2020‐04743), Eurostars (contract no. 2021‐03291), European Innovation Council (EIC) Accelerator (contract no 101188461), and the VINNOVA programme implementation of medical devices in healthcare (contract no. 2024‐01909). Multi4 Medical AB holds the patent and financed the project.

## Ethics Statement

The protocol for the *in vivo* animal studies was approved by the regional animal ethics committee, Gothenburg, Sweden (Dnr 2721/2019). The clinical study was approved by the Swedish Ethical Review Authority and the Swedish Medical Products Agency (5.1‐2023‐61 059) and was registered at ClinicalTrials.gov (NCT06136312).

## Supporting information


**Data S1.** Materials and Methods.

**Fig. S1.** Transportation of tissue biopsy through the instrument. After capture in the closed forceps, activation of a foot pedal drives the pump, moving the biopsy (dashed line) into the collection chamber (dotted square, enlarged in inset). The white arrow indicates the pump connection. The black arrow indicates the syringe port for local anaesthetic injection through the needle.

**Fig. S2.** Surgical treatment of a tumour using the Multi4 instrument. (a) Administration of local anaesthetics near the tumour using the injection needle (indicated by an arrow). (b) The biopsy forceps of the Multi4 instrument in an open position, ready to obtain tissue samples from the tumour. (c) Coagulation of the area following tumour resection.

**Fig. S3.** Flowchart outlining the healthcare process related to transurethral resection of the bladder (TUR‐B) in standard care compared with the procedure using the Multi4 instrument. In standard care (top panels), patients undergo preoperative visits approximately 1 week before surgery, followed by admission on the day of the procedure. The procedure is performed under sedation or spinal anaesthesia in the operating room, with postoperative observation, transfer to inpatient care and overnight hospitalization before discharge the following day. In outpatient TUR‐B (middle panels), the procedure is performed under similar anaesthetic conditions, with patients typically discharged the same day after observation at the outpatient clinic. In contrast, the Multi4 approach (bottom panel) eliminates the need for preoperative visits and hospitalization. Patients arrive shortly before the procedure, undergo local anaesthesia without urinary catheterization and have the biopsy resection completed in an outpatient setting. The patients can leave within minutes after the procedure, without the need for postoperative ward stay. The Multi4 pathway thereby streamlines TUR‐B management, reducing resource use and hospitalization compared with standard care.

**Fig. S4.** *Ex vivo* setup. (a) Schematic illustration of the *ex vivo* setup developed to use a bladder from pig slaughter waste for initial functionality testing of the Multi4 instrument. (b) The Multi4 instrument used through a cystoscope to take a biopsy from the bladder wall in the *ex vivo* setup.

## Data Availability

The datasets used and/or analysed during the current study are available from the corresponding author on reasonable request.
