# Supplementary material for: First‐in‐human outpatient management of recurrent NMIBC with the Multi4 instrument
Source: BJU Int. 2026 Sep 11;138(5):867–76. doi: 10.1111/bju.70445 (PMC13655195; doi:10.1111/bju.70445)
Supplement: Supplementary file 1 [file BJU-138-867-s001.docx]

**Supplementary Information**

**Materials and Methods**

***Ex vivo* functionality testing**

Fresh or thawed frozen urinary bladders were obtained from pig slaughter waste. Each bladder was mounted in a custom-designed bladder fixture isolating base that was electrically connected to the neutral electrode of an Electrosurgical Unit (ESU), as the Multi4 instrument uses monopolar electrosurgery. The outer surface of the bladder was surrounded by saline to facilitate electrical conductivity and simulate physiological conditions, while the bladder lumen was filled with sterile water to provide realistic distension during cystoscopic procedures.

This setup enabled full deployment and operation of the Multi4 instrument as if in a patient setting, including cystoscopic insertion, visualization, biopsy retrieval, electrosurgical cutting, coagulation, and fluid handling. All required components of the Multi4 instrument were integrated into the *ex vivo* platform, including the Multi4 instrument and pump, ESU, cystoscope with compatible connector, biopsy container, and fluid management components.

***In vivo* safety and functionality evaluation in pig**

Only female pigs were used in this study to avoid anatomical complications associated with the male urethra, which features a sigmoid flexure not present in humans and which hinders cystoscopic access. The pigs were housed together before surgery, the rooms had bedding consisting of wood shavings and straw, and the pigs were fed twice daily and had free access to water. Anaesthesia was induced via intramuscular injection of dexmedetomidine (30 μg/kg; Dexdomitor, Orion Pharma Animal Health), tiletamine (3 mg/kg), and zolazepam (3 mg/kg; Zoletil, Virbac). Animals were then intubated and maintained under inhalation anaesthesia using isoflurane (Attane vet, VM Pharma) at a concentration of 2.5–3.8%, corresponding to 1.3–1.5 minimal alveolar concentration (MAC). For perioperative analgesia, carprofen (4 mg/kg; Norocarp vet, N‐vet) and buprenorphine (0.03 mg/kg; Vetergesic vet, Orion Pharma Animal Health) were administered intravenously. Cystoscopy was performed by introducing a flexible cystoscope (Olympus VHA) through the urethra into the bladder and the Multi4 instrument was advanced through the working channel of the cystoscope. Visualization during cystoscopy was achieved using an Olympus CYF-VHA Cystoscope, including Olympus CV-170 Video processor and light source. Safety and functionality evaluation was performed *in vivo* on 13 pigs and biopsies were obtained from multiple sites within the bladder, including both individual sampling points and locations where 5-6 sequential biopsies were taken at the same site.

To ensure that repeated biopsies taken from the bladder tissue will not induce perforation of the bladder, repeated biopsies were taken at the same site until perforation. To monitor possible perforation, the bladder was filled with diluted contrast fluid (Urografin 76, Bayer). As control, a drop of the fluid was added to gauze and visualized using X-ray. A section was performed at *linea alba* and the bladder was exposed. The outer surface of the bladder was covered with gauze and analyzed for leakage of contrast fluid by x-ray after each biopsy. Perforation tests were performed at 5 different locations in different pigs. For reference, control biopsies were performed using conventional cold cup biopsy forceps (Karl Storz). To evaluate the hemostatic capacity of the biopsy technique, 10,000 IU of heparin (Leo Pharma) was administered intravenously prior to performing biopsies with both the Multi4 instrument and standard cold cup forceps. In addition, the injection needle function of the Multi4 instrument was assessed by injecting 3 ml of fluid into the bladder wall. The technique used corresponds to standard transurethral resection of the bladder, in which tumors are similarly fragmented to allow for evacuation.

**Health economic analysis**

A scenario-based health economic cost analysis was conducted to compare the standard TUR-B procedure with TUR-B using the Multi4 instrument in patients with recurrent NMIBC. The evaluation followed standard methodologies, including identification, quantification, and valuation of healthcare resources. The analysis adopted a healthcare provider perspective and included all direct medical costs related to a TUR-B episode, excluding follow-up or indirect societal costs. Unit costs were derived from the Cost Per Patient (CPP) database at the Sahlgrenska University Hospital (SU) for 2023–2024, supported by data from the Orbit surgical planning system and supplemented with expert opinion.

The standard care TUR-B involved a pre-operative visit, surgery under general or spinal anesthesia in an operating room, and potential post-operative hospitalization. In contrast, the Multi4 procedure was performed under local anesthesia during a single outpatient visit without the need for pre-operative consultations or inpatient care.

Estimated costs were calculated per patient and care contact. Scenario analyses were conducted to test variations in cost components (±20%) and to explore cost thresholds at which the Multi4 instrument would no longer be cost-saving. A regional analysis was also performed using epidemiological data from Region Västra Götaland (VGR) to assess potential savings from different levels of system adoption. The analysis was performed by Gothia Forum, a regional clinical research support organization independent of the device manufacturer and not involved in device development.

**Results**

***Ex vivo* functionality testing**

To simulate the clinical environment and systematically evaluate the usability and functionality of the Multi4 instrument under controlled conditions, an *ex vivo* porcine bladder model was developed (Supplement Fig 4.). Urinary bladders were obtained from slaughterhouse waste, eliminating the need for ethical approval. Prior to initiating *in vivo* studies, extensive testing was conducted using this model, resulting in more than 5,000 individual procedures. These assessments verified key functionalities of the instrument, including tissue cutting for biopsy retrieval and coagulation using monopolar diathermy to achieve hemostasis or simulate tumor ablation. The *ex vivo* model provided a reproducible and ethically sustainable platform that closely mimicked the anatomical and mechanical properties of the human bladder, thereby enabling reliable evaluation of device performance, procedural outcomes and tool-tissue interactions prior to clinical translation.

**Supplementary Figure Legends**

**Supplement Fig. 1 Transportation of tissue biopsy throught the instrument.** After capture in the closed forceps, activation of a foot pedal drives the pump, moving the biopsy (dashed line) into the collection chamber (dotted square, enlarged in inset). White arrow indicates pump connection. Black arrow indicates syringe port for local anaesthetic injection though the needle.

**Supplementary Fig 2. Surgical treatment of a tumor using the Multi4 instrument.** a) Administration of local anaesthetics near the tumor using the injection needle (indicated by an arrow). b) The biopsy forceps of the Multi4 instrument in an open position, ready to obtain tissue samples from the tumor. c) Coagulation of the area following tumor resection.

**Supplementary Fig 3. Flowchart outlining the healthcare process related to TUR-B in standard care compared with the procedure using the Multi4 instrument.** In standard care (top panels), patients undergo pre-operative visits approximately one week before surgery, followed by admission on the day of the procedure. The procedure is performed under sedation or spinal anaesthesia in the operating room, with post-operative observation, transfer to inpatient care and overnight hospitalization before discharge the following day. In outpatient TUR-B (middle panels), the procedure is performed under similar anaesthetic conditions, with patients typically discharged the same day after observation at the outpatient clinic. In contrast, the Multi4 approach (bottom panel) eliminates the need for pre-operative visits and hospitalization. Patients arrive shortly before the procedure, undergo local anaesthesia without urinary catheterization and have the biopsy resection completed in an outpatient setting. The patients can leave within minutes after the procedure, without the need for post-operative ward stay. The Multi4 pathway thereby streamlines TUR-B management, reducing resource use and hospitalization compared with standard care.

**Supplement Fig 4. *Ex vivo* setup.** a) Schematic illustration of the *ex vivo* setup developed to use bladder from pig slaughter waste for initial functionality testing of the Multi4 instrument. b) The Multi4 instrument used through a cystoscope to take a biopsy from the bladder wall in the *ex vivo* setup.
